# Incorporating BEAMing technology as a liquid biopsy into clinical practice for the management of colorectal cancer patients: an expert taskforce review

**DOI:** 10.1093/annonc/mdx501

**Published:** 2017-09-04

**Authors:** J García-Foncillas, E Alba, E Aranda, E Díaz-Rubio, R López-López, J Tabernero, A Vivancos

**Affiliations:** 1 Cancer Institute, University Hospital Fundacion Jimenez Diaz, Autonomous University, Madrid;; 2 Medical Oncology Unit, Regional University Hospital Virgen de la Victoria, IBIMA, Málaga;; 3 Biomedical Research Institute (IMIBIC), Hospital Reina Sofía, Faculty of Medicine, Universidad de Córdoba, Cordoba, Spain (CIBERONC);; 4 Research Institute IdISSC, Hospital Clínico San Carlos, Faculty of Medicine, Universidad Complutense de Madrid, Madrid, Spain (CIBERONC);; 5 Medical Oncology Department and Translational Medical Oncology Group, University Clinical Hospital & Health Research Institute (IDIS); CIBERONC, Santiago de Compostela, University School of Medicine, Santiago de Compostela, Spain;; 6 Medical Oncology, Vall d’Hebron University Hospital and Institute of Oncology (VHIO), Universitat Autònoma de Barcelona (UAM), Barcelona, Spain (CIBERONC);; 7 Vall d'Hebron Institute of Oncology (VHIO), Cancer Genomics Lab., Barcelona, Spain

**Keywords:** colorectal cancer, mutation testing, liquid biopsy, EGFR, tumor resistance

## Abstract

The importance of mutation identification for advanced colorectal cancer treatment with anti-epidermal growth factor receptor agents is well established. However, due to delays in turnaround time, low-quality tissue samples, and/or lack of standardization of testing methods a significant proportion of patients are being treated without the information that Kirsten rat sarcoma and neuroblastoma rat sarcoma (RAS) testing can provide. The detection of mutated circulating tumor DNA by BEAMing technology addresses this gap in care and allows these patients to receive international guideline-recommended expanded RAS testing with rapid turnaround times. Furthermore, the overall concordance between OncoBEAM RAS colorectal cancer testing and standard of care tissue testing is very high (93.3%). This article presents an overview of the clinical utility and potential applications of this minimally invasive method, such as early detection of emergent resistance to anti-epidermal growth factor receptor therapy. If appropriately implemented, BEAMing technology holds considerable promise to enhance the quality of patient care and improve clinical outcomes.

## Introduction

Colorectal cancer (CRC) is the third most commonly diagnosed neoplasm and the third leading cause of cancer death in the United States [1], whereas it is the most common cause of cancer death after lung cancer in Europe [2]. Approximately one-quarter of patients with CRC present with metastatic disease (mCRC) at diagnosis (synchronous disease), and ∼40% of patients develop metachronous metastases after treatment, contributing to the high mortality rate associated with this disease. Systemic therapy is the mainstay of treatment for mCRC, and there are currently several approved drugs for the management of mCRC, which include chemotherapy agents, small molecules, and monoclonal antibodies.

Anti-epidermal growth factor receptor (EGFR) targeted therapies, such as cetuximab and panitumumab, do not have universal efficacy. In mCRC, there is a high frequency of Kirsten rat sarcoma (KRAS) viral oncogene homolog mutations, which are responsible for resistance to these monoclonal antibodies [3]. Traditionally, prescribing anti-EGFR therapy required assessment of mutations in KRAS Exon 2, which occur in ∼40% of patients. Additional research has expanded the spectrum mutations in KRAS and neuroblastoma RAS (NRAS) viral oncogene homolog genes that predict a lack of efficacy to these treatments, including mutations in KRAS exon 3 (codons 59 and 61) and exon 4 (codons 117 and 146) and mutations in NRAS exons 2, 3, and 4 [4–8]. Accordingly, clinical practice guidelines in the United States and Europe have been updated to reflect the need for broader RAS testing, in order to refine the most appropriate patient population to receive anti-EGFR therapy [9–11].

Molecular analysis of genomic alterations has been traditionally performed on archival formalin-fixed paraffin-embedded (FFPE), but this procedure entails limitations in terms of poor quality of the extracted DNA and lack of standardization of testing methods. Moreover, tumor heterogeneity and clonal molecular evolution throughout therapy confound tissue sampling and there is no consensus as to whether analysis of the primary tumor is sufficient or whether a metastatic lesion should be studied in patients with metastases [12–14]. As recurring tissue biopsies are not routinely performed in patients with advanced, the use of circulating tumor DNA (ctDNA) to detect tumor-specific mutations has emerged as an important tool to inform clinical decisions according to tumor dynamics. This procedure is often referred to as a ‘liquid biopsy’ and represents an attractive strategy for better patient selection and treatment individualization throughout multiple lines of therapy [15].

## Concept and methodological features of liquid biopsy

Liquid biopsy allows the analysis of several blood-based biomarkers, i.e. circulating tumor cells, protein molecules, mRNA, microRNA, and cell-free DNA. Methodological limitations seem to be more important for CTCs than for cell-free circulating nucleic acids [16]. In fact, circulating DNA has been investigated for decades as a potential marker for screening, diagnosis, prognosis, and monitoring treatment response. Indeed, the release of DNA into the bloodstream from apoptotic or necrotic tumor cells has been reported in different neoplasms [17–19]. Bettegowda et al. showed that the frequency of cases with detectable ctDNA was proportional to the stage of the disease. Although ctDNA was detected in 100% of patients with stage IV malignancies including colorectal, bladder, and ovarian cancer the rate of detection of ctDNA in early-stage malignancies was ∼50% [20]. They also compared the quantities of ctDNA and CTCs in the same blood sample from patients with solid tumors and showed that the level of ctDNA was always higher than that of CTCs. In 13 of 16 patients, ctDNA levels were relatively high, whereas no CTCs at all could be detected [20].

One of the main advantages of ctDNA analyses is its high degree of specificity, since somatic mutations found in ctDNA are not present in normal cell-free DNA. However, the analysis of ctDNA is challenging and requires highly sensitive techniques. Advances in the pre-analytical stage can improve the performance of ctDNA detection, for example ctDNA should be extracted from plasma rather than serum due to its lower concentration of background wild-type DNA in plasma [21]. After years of investigation there are now several methods for detecting ctDNA, and platforms primarily based on digital PCR and next-generation sequencing (NGS) are widely used, though each has inherent advantages and disadvantages in terms of sensitivity, specificity, throughput, and breadth of mutational coverage (Table 1). Readers interested in further details on the principles and different characteristics of these techniques are invited to consult the following recent review [22]. Among them, an emulsion PCR-based technology platform known as BEAMing (Beads, Emulsions, Amplification, and Magnetics) is the first liquid biopsy test that has been clinically validated [20, 23–26] and is CE Mark’d (OncoBEAM RAS CRC test) for testing the RAS-mutation status in CRC patients (OncoBEAM RAS CRC IVD IfU).

**Table 1.** Overview of technologies used for detection of circulating tumor DNA (ctDNA)

Method of detection	Test	Detection limit (% ctDNA)	Advantage(s)	Limitation(s)
Digital PCR	Droplet-based digital PCR	∼0.01%	High sensitivity	Detection of limited genomic loci/single-nucleotide variants
	BEAMing		Ease of use (available kits)	
	Microfluidic digital PCR		Clinically validated	
Targeted deep sequencing (with NGS)	SafeSeq/TamSeq/Ion-AmpliSeq/Ontarget/CAPP-Seq	∼0.01–2.0%	High sensitivity	For selected alterations across targeted regions
			Relatively inexpensive	Need of assay personalization (except for CAPP-Seq)
Whole-genome sequencing (with NGS)	Digital karyotyping/PARE	∼1%	Broad application without personalization	Expensive
				Low sensitivity
Whole-exome sequencing (with NGS)	Currently for research purposes	∼5%	Broad application without personalization	Expensive
				Low sensitivity
				Lack of standarization

PCR, polymerase chain reaction; BEAMing, beads, emulsion, amplification, and magnetics; NGS, next-generation sequencing; SafeSeq, safe sequencing system; TamSeq, tagged amplicon deep sequencing; CAPP-Seq, cancer personalized profiling by deep sequencing; PARE, personalized analysis of rearranged end.

## OncoBEAM platform: analytical and clinical validity in CRC

The OncoBEAM platform addresses the technical challenge of identifying rare DNA molecules with enhancements to the PCR process known as BEAMing. DNA amplification is used to increase the quantity of DNA species of interest and facilitate measurement. Conventional PCR carries out one reaction per single sample and provides one signal. BEAMing involves partitioning of the PCR process into many individual reactions to provide high resolution detection of rare DNA sequences (e.g. ones having a RAS mutation). In performing BEAMing, each DNA molecule in a sample is amplified and converted into a single magnetic particle to which thousands of copies of DNA identical in sequence to the original DNA molecule are bound. The resulting magnetic particles (beads) are a one-to-one representation of the starting DNA molecules in a given sample and are compartmentalized into water-in-oil microemulsions such that each emulsion contains only one DNA molecule template. A subsequent amplification step is performed within each emulsion. Flow cytometry is then applied to assess the variation within the original population of DNA molecules. BEAMing is designed to assess hundreds of millions of individual DNA molecules with standard laboratory equipment. BEAMing can be used for the identification and subsequent quantification of cell-free tumor DNA molecules, which are differentiated from normal (wild-type) DNA by somatic mutations [27]. As a result, this technology is highly sensitive and reliably detects mutated ctDNA even when the mutation exists as a rare event, at a level as low as 0.01% [28].

The OncoBEAM assays are able to analyze hotspot mutations and the number of mutations detected varies per tumor type analyzed. The OncoBEAM RAS CRC Kit detects 34 mutations in codons 12, 13, 59, 61, 117, and 146 of the KRAS and NRAS oncogenes. For other relevant oncogenes, CRC guidelines recommend BRAF mutational analysis to be performed solely for prognostic stratification [29]. A second-generation version of the OncoBEAM CRC kit will incorporate BRAF in order to ensure patients with BRAF mutations are segregated from the RAS wild-type patients.

OncoBEAM RAS testing has been investigated in several head to head studies and these have demonstrated high concordance between blood-based testing versus standard tissue-based RAS testing methods [26, 30–32]. In particular, OncoBEAM was utilized to evaluate the concordance of RAS status between plasma and tissue in a large cross-section of mCRC patients [26]. In this evaluation, two geographically separated cohorts of CRC patients with metastatic disease from Germany and Australia were selected to represent the intended use population for anti-EGFR therapy, with plasma taken at the time of tissue biopsy or surgical resection. Tissue RAS status was evaluated according to the local standard of care and compared with plasma OncoBEAM RAS testing results. This initial study showed that the concordance between plasma RAS status determined by BEAMing versus that obtained by FFPE testing was 91.8% for stage IV CRC patients. A larger performance evaluation from six centers in Europe showed that the overall concordance between OncoBEAM RAS CRC testing and standard of care tissue testing for 238 patients was 93.3% [33]. The RAS mutation status determined by OncoBEAM from plasma versus the tissue reference method is summarized in Table 2. Plasma RAS mutations were found in 112/121 KRAS mutant cases determined by tissue-based testing (92.6% of positive percent agreement) and no RAS mutations were found in 110/117 not mutant cases according to tissue testing (94% of negative percent agreement. Overall, RAS mutations were detected in 51% of tumor-tissue samples and in 50% of plasma samples [33]. The frequency of RAS mutations in patients investigated in this study was in agreement with the results of other groups performing expanded RAS analysis [34]. All this evidence provides a high level of confidence that the clinical performance of plasma RAS testing using OncoBEAM RAS is comparable with FFPE tissue testing and can be useful in a clinical setting to select advanced CRC patients for anti-EGFR therapy.

**Table 2.** Concordance of RAS mutation status: plasma ctDNA versus tumor tissue analyses

	Tumor-tissue RAS result						
	RAS	Mutant	WT	Total	PPA (95% CI)	NPA (95% CI)	OPA (95% CI)
**Plasma ctDNARAS result**	Mutant	112	7	119	100×112/121=92.6% (86%, 96%)	100×110/117=94.0% (88%, 97%)	100×222/238=93.3% (89%, 96%)
	WT	9	110	119			
	Total	121	117	238			

RAS, Fisher’s exact test was used to test for a relationship between RAS mutation-positive results in plasma versus tissue samples (positive percent agreement, PPA), WT results in plasma versus tissue samples (negative percent agreement, NPA), and the combination of RAS mutation-positive and WT results in plasma versus tissue samples (overall percent agreement/concordance, OPA). Data from reference [33].

The minimal differences in RAS mutation status between plasma and tissue may be attributed to intra- or inter-tumor molecular heterogeneity or variability in tissue techniques. While a false negative result (no mutation detected in a patient with a RAS mutant tumor) in either tissue or plasma may lead to the inappropriate assignment of first-line treatment anti-EGFR therapy (with the risk of detrimental outcome with anti-EGFR exposure, the risk of toxicity and/or allergic reaction and the high expenses associated with these agents), this risk is largely mitigated by the high frequency of radiographic surveillance in mCRC patients undergoing therapy. A false positive result (mutation detected in a patient that does not have a RAS mutation) may lead to a patient forgoing first-line anti-EGFR therapy and receiving a less effective chemotherapy. This risk may be mitigated by the longitudinal evaluation of plasma RAS status during therapy in order to inform subsequent treatment lines [32]. The evaluation of the outcomes of false positive and negative cases with OncoBEAM RAS testing is an area of active investigation. As a more comprehensive elucidation of ever-changing tumor dynamics and host biological influences emerges, this information will lead to a better understanding of biological factors influencing ctDNA testing and subsequent implementation into clinical practice.

## BEAMing advantages and current usefulness

The current standard of care for CRC patients involves tissue mutation testing to determine whether a patient will benefit from the administration of anti-EGFR therapy. Blood-based RAS mutation testing should address the following unmet needs associated with tissue testing:

- Delayed processing: Plasma testing is favorable in newly diagnosed patients or when a patient’s mutation status is unknown and expedited results are required for therapy selection (reduction of turnaround time to <7 days). Recently, a quality survey found that half of all European participating laboratories exceeded the required turnaround time of 14 days for complete RAS tissue-based testing [35]. These delays in processing tissue may prohibit the timing of molecular testing results being available at the time of a patient’s visit, thus delaying the prescription of optimal therapy.

Invasive biopsies: Obtaining a blood sample is minimally invasive to the patient compared with obtaining a tissue sample and provides an alternative when tumor tissue is insufficient for molecular testing. Need of repeated biopsies in case of metastatic disease: In a patient with recurrent disease, archival tissue may not reflect current mutational status and it is common practice to not perform a tissue biopsy at time of recurrence. A liquid biopsy test can provide real-time information about the current mutational status of the primary tumor and can serve as an alternative to archival tissue, which suffers from degradation and may reflect an ‘archival’ mutation profile that may not be not representative of the current status [36]. Additionally, a systemic assessment of tumor mutational status is not practical or feasible in a patient with metastatic disease. Evidence of molecular heterogeneity among primary and metastatic sites [14, 37] suggest that a ctDNA assessment of the mutation status of systemic tumor burden can more precisely guide targeted systemic therapy [14].

When compared with other platforms including NGS, OncoBEAM assays detect mutations at a higher level of sensitivity with a focus on clinically actionable gene mutations. While NGS does have the capability to detect additional gene mutations, the clinical utility of these additional variants is often not well established or aligned to an approved therapeutic; an additional limitation is its low sensitivity. OncoBEAM blood-based assays offer rapidity of results compared with NGS, with OncoBEAM RAS testing delivering results within 2–3 days, a critical advantage when treating patients with advanced or progressing disease. Moreover, a recent review focused on different technologies for molecular classification of cancers established that BEAMing is associated with the lowest costs comparing with other digital PCR or NGS techniques [22]. Since OncoBEAM RAS test analyzes an expanded panel of 34 RAS mutations in KRAS and NRAS, it provides RAS testing in accordance with the current standard of care molecular testing guidelines as defined as the ESMO and NCCN committees.

## BEAMing future applications

### Real-time monitoring of treatment

Minimally invasive diagnostic assays performed by BEAMing are ideally suited for disease monitoring throughout therapy. Various studies have demonstrated that ctDNA measurements can be used to reliably monitor tumor dynamics in subjects undergoing surgery or chemotherapy [38, 39]. In these studies, ctDNA levels were compared with imaging and biomarkers such as carcinoembryonic antigen (CEA) in predicting response to treatment. Results demonstrated that BEAMing was more sensitive than imaging, CEA, or total DNA to provide an earlier indication of response to treatment. This is further supporting evidence to show that not only does ctDNA levels correlate with changes in tumor burden, they provide a more immediate and sensitive measure of response than either imaging or CEA [28, 39]. Vidal et al. [32] examined the utility of OncoBEAM RAS CRC ctDNA testing to monitor the efficacy of response to treatment taking serial blood draws from 21 patients with baseline RAS mutations undergoing systemic therapy. Analysis of RAS ctDNA at the time of a first CT scan (8–12 weeks of treatment) revealed a dramatic decrease in plasma RAS mutant allele fraction (MAF) in responding patients with a median of 100%. MAF percentage of change was significantly lower in patients that progressed at first evaluation of response compared with patients with clinical benefit (132% increase versus 99% reduction, respectively, *P* = 0.027). The authors concluded that RAS plasma mirrored clinical and radiological response to chemotherapy drugs and was an early predictor of response [32].

### Detection of resistance mechanisms

A blood-based approach enables detection of emergence or disappearance of genetic mutations linked to resistance or susceptibility to targeted therapies [24]. Emergence of RAS mutations is a frequent mechanism of resistance in mCRC patients treated with anti-EGFR therapy. A recent study utilizing the OncoBEAM RAS assay provides preliminary evidence to support the role of monitoring emerging RAS in CRC patients receiving anti-EGFR therapy [40]. In this study, 62 of 70 (89%) of mCRC patients who initially responded to anti-EGFR therapy and chemotherapy were found to develop resistance. At the time of resistance, acquired mutations in KRAS were detected by BEAMing in the plasma of 27/62 (44%) patients. In order to evaluate whether newly detected KRAS mutations were already present in treatment-naïve primary tumors as undetectable low frequency clones or were truly acquired mutations, the investigators utilized the OncoBEAM RAS assay on the original tumor tissue to re-analyze archival samples from 20 of 27 patients for traces of KRAS clones. This analysis revealed that seven (35%) patients had low-frequency KRAS mutations and that overall, these seven patients had a poorer prognosis than those determined to be truly KRAS wild type (median progression-free survival: 3.0 versus 8.0 months, *P* = 0.0004) [40]. In another study [8], researchers conducted a *post hoc* investigation of patients enrolled in the phase III CRYSTAL study in order to determine the treatment effects of cetuximab plus FOLFIRI versus FOLFIRI alone for patients whose tumors had mutations in one of the less common RAS mutations (located in KRAS codons 61, 117 and 146, or NRAS codons 12, 13, and 61). Mutational status for an additional 26 RAS mutations was determined by the OncoBEAM RAS assay for these patients. Using a 5% mutant/wild-type cutoff, an additional 63 patients (14.7%) were classified as RAS mutant positive; 86 patients (20%) were identified when a less stringent cutoff ≥ 0.1% mutant/wild-type sequences was used. When considering efficacy outcomes between treatment groups, there was no clear benefit for the addition of cetuximab to FOLFIRI treatment in patients with either ≥5% RAS mutant/wild-type cutoff. However, patients with a RAS mutant allele fraction <5% were able to derive benefit from the addition of cetuximab to FOLFIRI [8]. The clinical application of OncoBEAM RAS CRC assay for monitoring of acquired resistance to anti-EGFR therapy in routine clinical practice has been recently evaluated by Vidal et al. [32]. Emergence of RAS mutations was detected in 7/18 patients (39%) showing disease progression after an initial complete response, partial response or stable disease for >16 weeks, and in three cases, different RAS mutations were concomitantly detected [32]. Moreover, Toledo et al. [41] performed a prospective validation of the BEAMing technique to monitor newly diagnosed KRAS wt mCRC patients who received a standard FOLFIRI-cetuximab regimen. They showed that the patients who initially responded to anti-EGFR therapy but later acquired resistance presented intermediate and gradually increasing levels of circulating mutant alleles, whereas patients with long-term responses maintained a wt circulating status throughout the anti-EGFR therapy. As data continues to emerge showing that the identification of RAS mutations in the plasma of relapsed patients indicates resistance to anti-EGFR therapy, regular analyses could inform clinical decision making and may offer patients the opportunity to benefit from therapies designed to overcome resistance, which in every instance is a more cost-effective approach compared with tissue-based clinical management.

### Early detection of relapse and/or residual disease

The potential of BEAMing technology in this regard was demonstrated in a proof of concept study that provides insight into the utility of ctDNA for monitoring treatment response and predicting disease recurrence following surgery [23]. In this study, mCRC patients were followed throughout both surgery and systemic treatment. A total of 18 patients underwent 22 surgeries of which 17 were complete tumor resections and 5 were incomplete resections. A BEAMing assay specific to the molecular profile of each patient was designed in order to perform plasma mutation assessments. Plasma samples were collected from 18 patients on the date of surgery, 24-h post-surgery, and then at regular intervals between 13 and 56 days. ctDNA levels decreased dramatically in all patients after surgery but were detectable in the first follow-up visit (within 13–56 days) in 16/20 instances (plasma samples were only available in 20 instances—not 22). In all but one of these instances the patients’ diseases recurred. In contrast, in 4 patients in whom mutant DNA was undetectable, no recurrence occurred [23]. The pilot study of Misale et al. [39] showed that emergence of KRAS mutations was detectable in plasma as early as 10 months before the documentation of disease progression by radiological assessment. In line with these results, ctDNA-based detection preceded clinical detection of metastasis in 86% of breast cancer patients with an average lead time of 11 months (range 0–37 months), whereas patients with long-term disease-free survival had undetectable ctDNA postoperatively [42].

A pressing question is how often to perform a liquid biopsy [8], while this is not yet established, the most practical frequency appears to be every 4–6 weeks interspersed and/or in conjunction with radiological scans. Serial ctDNA measurements could complement routine imaging-based assessments in evaluation of disease bulk [43], response to chemotherapeutic agents, re-challenge with anti-EGFR therapy [44], and detection of residual disease after surgical resection of the tumor [45]. In the last case, this approach could aid to select treatment strategies in patients with residual disease that could benefit from adjuvant chemotherapy and intensive surveillance.

### Early detection of cancer

It is worthwhile to highlight the potential application of OncoBEAM RAS testing as a screening tool for pre-neoplastic lesions or localized neoplastic disease. Bettegowda et al. [20] showed that ctDNA is more readily detected in the blood of patients with more invasive tumors rather than earlier tumors, but even so the sensitivity for detection was 50% in early-stage disease. However, only RAS mutant patients would benefit from the use of this test as screening method.

## Concluding remarks

The clinical utility of a diagnostic test pertains to the ability of the test to provide new information that leads to a clinical benefit. The importance of RAS testing in mCRC patient selection for anti-EGFR therapy is well established, but due to known limitations of tissue-based testing and delays in clinical turnaround time, a significant proportion of patients are being treated or receiving delayed treatment without the information that RAS testing can provide. The clinical utility of the OncoBEAM RAS test allows patients to benefit from international guideline-recommended expanded RAS testing with rapid turnaround times.

OncoBEAM RAS testing has also potential utility in monitoring patients who have relapsed on treatment with anti-EGFR therapy. Identification by blood-based OncoBEAM testing of any RAS mutation in relapsed patients may be indicative of emergent resistance to anti-EGFR therapy, providing insight into the timing of subsequent lines of therapy. The high degree of concordance of RAS testing results generated by blood-based OncoBEAM RAS testing versus standard tissue testing methods supports the conclusion that detection of RAS mutations in the blood with BEAMing may be a useful replacement to tumor testing. OncoBEAM RAS testing also makes possible to examine a minimally invasive method for detecting early resistance to anti-EGFR therapy. All these features represent a clear benefit to patient care. Incorporation of the OncoBEAM RAS into clinical practice is therefore likely to add precision and provide cost-effective management by individualizing treatment plans for the CRC patient. 
